# Two Machine Learning Models to Economize Glaucoma Screening Programs: An Approach Based on Neural Nets

**DOI:** 10.3390/jpm15080361

**Published:** 2025-08-07

**Authors:** Wolfgang Hitzl, Markus Lenzhofer, Melchior Hohensinn, Herbert Anton Reitsamer

**Affiliations:** 1 Department of Ophthalmology and Optometry, Paracelsus Medical University, University Hospital Salzburg, Muellner Hauptstrasse 48, 5020 Salzburg, Austria; m.lenzhofer@salk.at (M.L.); m.hohensinn@salk.at (M.H.); h.reitsamer@salk.at (H.A.R.); 2 Research Program Experimental Ophthalmology and Glaucoma Research, Paracelsus Medical University, Muellner Hauptstrasse 48, 5020 Salzburg, Austria

**Keywords:** glaucoma, machine learning, neural network, screening, prediction

## Abstract

**Background**: In glaucoma screening programs, a large proportion of patients remain free of open-angle glaucoma (OAG) or have no need of intraocular eye pressure (IOP)-lowering therapy within 10 years of follow-up. Is it possible to identify a large proportion of patients already at the initial examination and, thus, to safely exclude them already at this point? **Methods**: A total of 6889 subjects received a complete ophthalmological examination, including objective optic nerve head and quantitative disc measurements at the initial examination, and after an average follow-up period of 11.1 years, complete data were available of 585 individuals. Two neural network models were trained and extensively tested. To allow the models to refuse to make a prediction in doubtful cases, a reject option was included. **Results**: A prediction for the first endpoint, ‘remaining OAG-free and no IOP-lowering therapy within 10 years’, was rejected in 57% of cases, and in the remaining cases (43%), 253/253 (=100%) received a correct prediction. The second prediction model for the second endpoint ‘remaining OAG-free within 10 years’ refused to make a prediction for 46.4% of all subjects. In the remaining cases (53.6%), 271/271 (=100%) were correctly predicted. **Conclusions**: Most importantly, no eye was predicted false-negatively or false-positively. Overall, 43% all eyes can safely be excluded from a glaucoma screening program for up to 10 years to be certain that the eye remains OAG-free and will not need IOP-lowering therapy. The corresponding model significantly reduces the screening performed by and work load of ophthalmologists. In the future, better predictors and models may increase the number of patients with a safe prediction, further economizing time and healthcare budgets in glaucoma screening.

## 1. Introduction

Glaucoma is a leading cause of irreversible blindness [1]. Worldwide, it is estimated that about 66.8 million people have visual impairment caused by glaucoma, with 6.7 million suffering from blindness. Intraocular pressure (IOP) is the most important—because it is modifiable—risk factor for the development and progression of this disease [2]. Glaucoma is mostly asymptomatic until late in its course, when visual problems arise [3]. However, with early treatment, it is often possible to protect the eyes against serious vision loss [2]. Therefore, early diagnosis should be targeted. Due to the lack of symptoms in the early disease stage of glaucoma in most patients, this can only be achieved through screening programs. The most common subgroup of glaucoma in Europe is open-angle glaucoma (OAG). It is well known in glaucoma research that age, IOP, PEX, and topographic disc parameter are univariable risk factors for predicting OAG. 

A major goal in glaucoma research is to combine these risk factors in a multivariable fashion and to make predictions on a personalized level. Machine learning algorithms such as neural networks can personalize diagnosis for patients on an individual level, although several issues remain unresolved [4,5,6]. Because no such tools are currently in use that predict 10-year occurrence of OAG, we investigated to what extent a reliable prediction is feasible. 

Several univariable risk factors for developing OAG are well known, such as age [4], increased IOP [2], PEX [7,8,9], and topographic disc parameters such the cup-to-disc ratio (C/D) [10,11]. Increased age, IOP, and cup-to-disc ratio, as well as PEX, are positively related to an increased risk of OAG. For example, age is an important risk factor for developing OAG; however, it is still unclear precisely how it affects the progression of OAG over time, particularly in relation to and in combination with other risk factors. Even if all these univariable risk factors are identifiable through a single examination at one point in time, it is difficult to diagnose OAG in certain cases, so why should we even consider attempting to make a prediction over a long period of 10 years? 

There are mainly three reasons why models with a reject option should be used: (1) It is well known that patients with no or a small number of risk factors are highly likely to remain OAG-free over a long period. For example, subjects aged ≤ 50 years old with an IOP ≤ 18mmHg and a cup-to-disc ratio (C/D) ≤ 0.2 are highly likely to remain OAG-free over many years [3,10,11]. Thus, the basic idea is to identify a—hopefully as large as possible—subpopulation at the initial examination that will remain OAG-free at the 10-year follow-up point. (2) Studies have demonstrated that 10-year OAG incidence is quite low, ranging from 0.7 to 2.2% [12,13]. Hence, most patients remain OAG-free over 10 years, which makes predicting OAG freeness easier compared to incidences around 50%. (3) The third, and by far the most important, reason is related to the so called ‘reject option’ of the model. It is definitely not necessary to identify every single eye that remains stable. Usually, one expects that a model will make a prediction for every single patient. However, to reduce the screening time, it is sufficient to identify a preferably large subpopulation of patients with eyes that remain stable. If the prediction is too difficult and the accuracy of the prediction model is ‘in doubt’, it is still better to refuse to make a prediction instead of making a false-positive or false-negative prediction. Patients who do not receive a prediction are sent to the glaucoma specialist as usual. This so-called ‘reject option’ is crucial and a highly desirable advantage, because the models are not ‘forced to make a prediction’. In other words, if the model has enough evidence, then it makes a prediction; otherwise, it refuses to make a prediction and asks the glaucoma expert to make the final decision (or to make no prediction at all). There is a cost to developing this model, because we have to accept that a large number of eyes do not receive a prediction. On the other hand, the reject option substantially increases the accuracy of the model for those patients who receive a prediction. This allows for identifying a large number of patients for whom the corresponding eye can safely be excluded, and, thus, much time and effort in screening studies can be saved. All definitions, endpoints, methods, and results refer to right eyes, as machine learning algorithms are expected to clearly distinguish between right and left eyes. New models for left eyes can be trained and tested using the same methods.

This study will answer the following questions by considering the principle of very high accuracy: (1) Which of these endpoints shows reasonable results? (2) How good are the performances of the neural network models in terms of accuracy? How many patients receive a false-negative or false-positive prediction? (3) To what extent do these models help to reduce daily work load, i.e., how many eyes receive a prediction, and in how many cases is the algorithm in doubt? (4) How can these approaches be improved in the future? 

## 2. Materials and Methods

The Salzburg-Moorfields-Collaborative-Glaucoma Study (SMCGS) began in 1996 and is part of a large 25-year glaucoma blindness prevention program in Austria, Europe. The SMCGS is a retrospective monocentric population-based longitudinal cohort study. This study mainly provides information for and identifies glaucoma suspects, offering timely and adequate treatment in clear-cut cases of glaucoma.

### 2.1. Definition of Definite OAG

We defined glaucoma as a progressive optic neuropathy leading to the loss of retinal nerve fibers and glia cells, with characteristic alterations on the optic nerve head and with or without corresponding functional alterations in visual field examination [14]. The following parameters were used here: (a) Optic nerve head (ONH)—notching, rimming, arbitrary chosen subjective cup-to-disk (C/D) ratio of 0.45 or more, or C/D asymmetry between the two eyes >0.3 not explainable through other diseases. (b) Visual field—glaucomatous visual field defects corresponding to optic disc changes. (c) Open anterior chamber angle [14]. Inclusion criteria: age ≥ 40 years, no OAG or other type of glaucoma, best spectacle-corrected visual acuity >6/9, refractive range ranging from −6.00 to +4.00 diopters with a difference in refraction between both eyes <3.00 diopters. Exclusion criteria: pseudophakia, IOP-lowering therapy at the initial examination, and eye diseases potentially leading to visual field defects or secondary increases in intraocular pressure, contraindication against beta blockers, systemic corticosteroid therapy, or pregnancy. 

During the study’s duration of more than 27 years, up to 24 ophthalmologists and 7 ophthalmic assists specially trained in using the study protocol performed ophthalmic examinations and graded each patient. SMCGS data collection started on the 1st December 1996. Baseline: The ophthalmic assistants were responsible for oral education and SMCGS, questionnaire interviews, best corrected visual acuity (BCVA) measurements with the Early Treatment Diabetic Retinopathy Study (EDTRS) chart, subjective refraction, visual field examination (VF; Motion-Sensitivity Test (MST), Humphrey Frequency Doubling Technology Perimetry (FDT, Humphrey FDT matrix, Humphrey Instruments Inc., San Leandro, CA, USA), Humphrey Field Analyzer II or II-I [HFA, Humphrey Instruments Inc., San Leandro, CA, USA]), central corneal thickness (CCT) with ultrasound pachymetry (Pocket II, Quantel Medical, Cournon d’Auvergne Cedex, France) and structural imaging (confocal scanning laser ophthalmoscopy was carried out using TopSS^®^ (Fa. LDT, San Diego, CA, USA)), which refers to a scanning laser tomography technique for analyzing the eye, specifically the optic nerve head. This method uses scanning laser tomography to depict the top surface of the optic nerve, allowing for detailed analysis of structural changes. Subsequently, all subjects underwent a standardized eye examination with intraocular pressure (IOP) assessment in miosis and mydriasis through Goldmann applanation tonometry (Haag Streit, Koeniz, Switzerland), gonioscopy (to exclude angle-closure), slit lamp anterior segment examination, and dilated fundus slit lamp biomicroscopy for optic disc assessment using a Volk 90 D lens, including subjective C/D. PEX was diagnosed during the initial clinical examination in the glaucoma screening ambulance using a slit lamp. Recorded TopSS^®^ values included the C/D ratio, total contour area, effective area, neuroretinal rim area, half-depth area, half-depth volume, and volume below. If results were abnormal or inconclusive during the VF-examination at baseline, a second confirmation test was performed for clarification. 

### 2.2. Definition of Endpoints

We defined two different endpoints: E1—composite endpoint, i.e., freeness of OAG and no IOP-lowering therapy; E2: freeness of OAG (with or without IOP-lowering therapy). If a patient (1) still wants to join the screening program, (2) agrees to receive IOP-lowering therapy if necessary, and (3) is only interested in determining whether OAG will develop, then E2 is best. E1 is suitable for informing and safely excluding the patient from a screening program. Here, we want to show that for E1 and E2, ocular hypertension (OHT) might develop (in the case of E2, with a possibly necessary IOP-lowering treatment), but OAG will not develop.

Age, IOP [2], PEX [7,8,9], and 8 optic nerve head parameters (TopSS^®^) were used as continuous predictors for E1 and E2. 

#### 10 Years Follow-Up

Patients free of glaucoma (OAG or another type of glaucoma) at the baseline visit received follow-up only. All examinations were carried out the same manner as at the baseline examination. According to the results of the baseline examination, patients were also scheduled for interim visits in the first decade. Complete data for the baseline visit and the 10-year visit were used to train and validate the machine learning models. 

### 2.3. Statistical Methods and Model Development

Data were cleaned for inaccurate or missing data, resulting in data for 585 patients, with full records of each predictor and the outcome variable. Univariable logistic regression models were used to estimate the odds ratios of each individual predictor with 95% confidence intervals.

### 2.4. Machine Learning Algorithms, Training and Testing

Multilayer perceptron neural networks, nearest-neighbor classifiers, Bayes classifiers, random forest models, logistic regression models, and support vector machines were trained and tested. 

### 2.5. Data for Model Training and Testing

The full sample (n = 585) was randomly split into a training sample (n = 486) and a test sample (n = 117). No data from the test sample were used for model training. All neural networks were first trained and 10-fold cross-validated in a training sample (n = 468) and finally tested in an independent randomly selected test sample (n = 117) to ensure generalization to new, previously unseen eyes.

### 2.6. Methods for Model Training

#### 2.6.1. Neural Nets

Adaptive moment estimation was used as a stochastic gradient descent method. To mitigate overfitting, an early stopping approach and 10-fold cross-validation were used. L2-regularization techniques were also used. Additionally, early stopping, batch normalization layers, and L2-regularization techniques were used. The batch size was 64 and the maximal number of training rounds was 5000 (Table S1). The network architecture is illustrated in Figure S1.

#### 2.6.2. Other Models

All other models were trained according to the default settings of MATHEMATICA [15]. 

### 2.7. Feature Selection

Potential risk factors were selected as input variables, which are listed in Table 1. Although some of these risk factors were not significant, after applying a genetic algorithm and the reject option (as described in the next subsection), these predictors were still useful for limiting the number of rejected cases.

### 2.8. Reject Option

From a clinical point of view, most importantly, we wanted to avoid making false-negative predictions for both endpoints. This means that E2 would predict that the patient will not suffer from OAG within 10 years, even though they may develop OAG within this period. For E2, a false-negative prediction may lead to irreversible eye damage or even blindness. For E1, the same scenario would occur, and no medication would be administered, which could also result in OAG or blindness. The predictors, however, did not have enough predictive power to achieve this goal for the whole cohort. Thus, it was reasonable to allow the algorithms to apply a ‘reject option’ in doubtful cases [16]. This means that 2 cut-offs for the posterior probabilities, instead of 1 cut-off, were first chosen in the training sample and then applied and tested in the test sample. Finally, to assess model performances, the percentages of predicted subjects and total correct predicted cases were computed in the training and test samples and compared to determine whether the networks generalized well to new, previously unseen data.

The advantage of this approach was that it minimized the number of false-negative and false-positive predictions. This important advantage came at a cost, as a large number of cases did not receive a prediction. We recommend that patients who do not receive a prediction should still join a glaucoma screening program to benefit from expert medical knowledge.

All analyses were carried out using STATISTICA 13 [17] and MATHEMATICA 13, developed by Wolfram Research, Inc., Champaign, IL (2023) [15]. 

## 3. Results

In the original screening study, 6889 subjects were screened and 976 subjects were examined ten years later. Finally, after applying all inclusion and exclusion criteria and removing all missing data, 585 right eyes with complete data were included.

### 3.1. Demographic Data at Baseline

An overview is given in (Table 2).

### 3.2. Overview of Demographic Data at 10-Year Follow-Up

Within our dataset, OAGs included 6 primary open-angle glaucoma (1.03%) cases, 3 pseudoexfoliation glaucoma (0.51%) cases, 0 pigmentary glaucoma (0%) cases, and 12 normal tension glaucoma (2.05%) cases. A total of 41 of 585 eyes had OAG or received at least one or more types of IOP-lowering eye therapy within 10 years (E1), corresponding to an average 10-year incidence of 7% (5.1–9.4%). Differences between those who fulfilled E1 or not and the corresponding univariable prediction power (odds ratios) levels of possible candidate predictors are given in Table 1.

An overview of the performance accuracies of the machine learning models is given in Table 3, while the advantages and disadvantages of using the selected neural network models with both E1 and E2 are given in Table 4. The neural network models had the smallest percentage of unpredicted patients compared to the classical machine learning algorithm. An overview of the model performance with various combinations of cut-offs for endpoint E1 is given in Table S2. The logistic regression model (classical approach) also achieved 100% NPV; however, the number of unpredicted patients was considerably larger at 69.2%. The models M1 and M2 demonstrated excellent results, with a sufficiently large proportion of eyes receiving a prediction. Most importantly, no eye was predicted false-negatively or false-positively.

### 3.3. Illustration of the Models Using Real Data

To better illustrate the models, typical data used in a glaucoma screening setting are provided at the initial examination, illustrating the predictions, decisions, and observations made by M1 for E1 (Table 5). On the left side, we see the predictors evaluated during the initial examination: age, IOP, effective area, neuroretinal rim area, volume below, half-depth area, half-depth volume, C/D, and PEX. On the right side, we see the predictions for M1 for E1 during the initial examination and corresponding observations of both endpoints made at the 10-year follow-up. Eyes highlighted in green were identified via the prediction model, and every single eye was correctly predicted. Eyes highlighted in gray did not receive a prediction, because the algorithm believed that their status was ‘in doubt’ and refused to make a prediction (i.e., the corresponding eye was ‘filtered out’). For E1, 332 eyes (57%) were filtered out and did not receive a prediction, while 253 (43%) of all eyes received a prediction. In summary, all 253 eyes were correctly predicted (100%), meaning that no eye was predicted false-negatively or false-positively.

### 3.4. Illustration for the Need for a Reject Option

**Figure 1:**
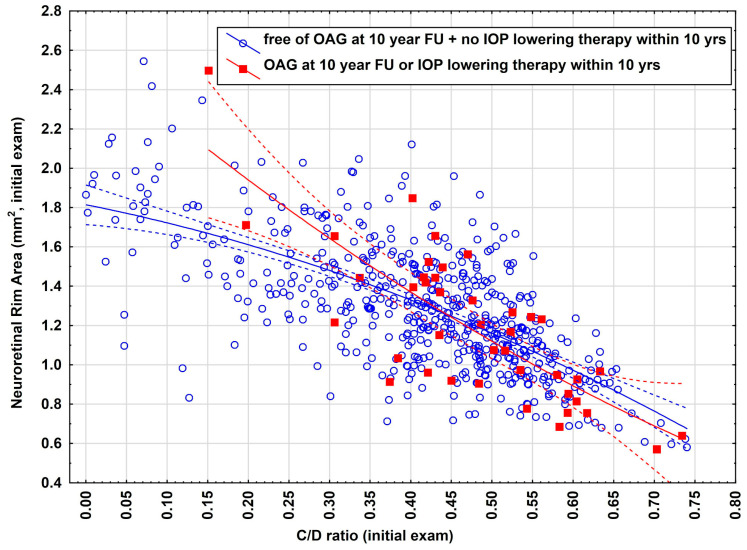
Scatterplot of C/D ratio and neuroretinal rim area (mm^2^) of 585 eyes suggesting the need of a reject option. All eyes were OAG-free without receiving IOP-lowering eye therapy at the initial exam. Red dots indicate eyes fulfilling endpoint E1 (freeness of OAG + no IOP-lowering therapy within 10 years of FU) at the 10-year FU, blue dots indicate eyes which do not fulfill endpoint E1 at the 10 yr FU. Eyes with values in the left part of the figure (‘low risk region’) are very likely to remain OAG-free and having no IOP-lowering therapy within 10 years whilst eyes in the right region (‘high risk region’) are at high risk for developing a OAG or will receive an IOP-lowering therapy within 10 years of FU. It is precisely the main task of neural networks to identify regions of low risk in the space of all input variables (including age, IOP, optic nerve head parameters and PEX), simultaneously.

To better understand both why a large number of eyes were filtered out and the difficulty of predicting E1, we provide deeper insights how close both data clouds (E1, yes/no) closely stick together and illustrate the overlap between both data distributions (Figure 1). As indicated, there is a large overlap between both distributions, indicating the difficulty of identifying eyes for which accurate predictions can be made and eyes that must be rejected during the initial examination for making predictions for the future.

## 4. Discussion

This study provides a completely new and safe approach for identifying a large subpopulation of eyes remaining free of open-angle glaucoma without needing IOP-lowering therapy within 10 years. The most important benefit of M1 is that 43% of the eyes in the screening population could safely be excluded already at the initial examination, i.e., no predicted eye received a false-positive or false-negative prediction. It is important to emphasize that this accuracy could only be achieved by allowing the algorithm to refuse to make a prediction, occurring in 57% of eyes. Therefore, there is no need to further examine eyes that receive a OAG screening prediction within the next 10 years, saving corresponding time, effort, and costs. This implies that these eyes are no longer within a screening program and, thus, will not receive IOP-lowering therapy. This was a key point and crucial for explaining not only why E1 focuses on OAG but also why these eyes do not receive IOP-lowering therapy. 

### 4.1. Discussion of Model M1

In this model, the patient will receive a positive response if they not only remain OAG-free over 10 years but also do not require IOP-lowering therapy, which is an important form of subjective relief for the person.

### 4.2. Discussion of Model M2 

**Figure 2:**
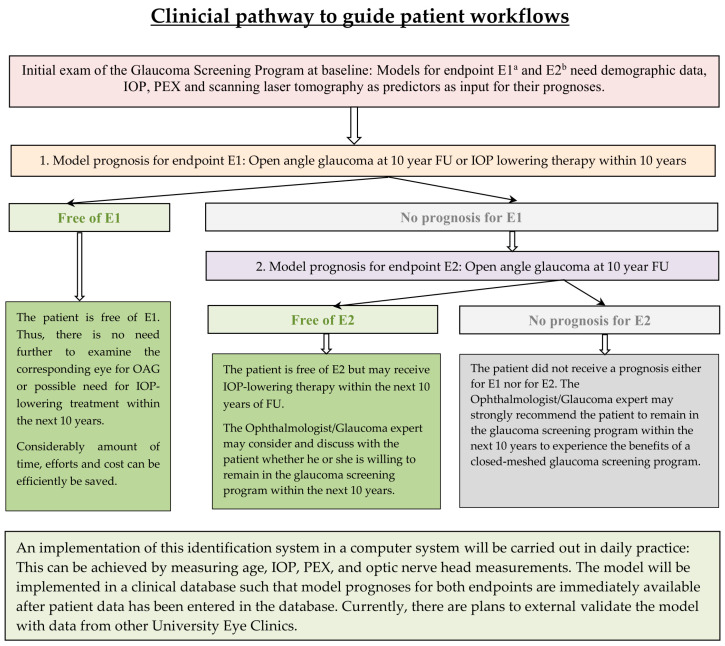
Clinicial pathway to guide patient workflows. a: Endpoint 1; b: Endpoint 2.

The patient receives a positive response if they remain OAG-free over 10 years. This model is the right choice for patients who are willing to remain in a glaucoma screening program. In this case, the eye may still receive IOP-lowering therapy if necessary. Therefore, E2 was defined as remaining OAG-free (with adequate IOP-lowering therapy if ocular hypertension arises). IOP-lowering therapy was not included compared to E2. A detailed overview of the clinical pathway used to guide the patient workflow is given in Figure 2.

### 4.3. PEX as Risk Factor and How It Was Handled by the Models

Additionally, we analyzed how the models handled patients with PEX. There were 570 eyes without PEX, and 44% received a prediction. All predictions were correct. A total of 15 eyes had PEX, and the algorithms refused to make a prediction in all 15 eyes. This suggests that PEX is a risk factor that deserves special attention from glaucoma experts.

### 4.4. Implication for Practical Purposes

This identification system can be implemented in a computer system in daily practice: This can be carried out by measuring age, IOP, PEX, and optic nerve head measurements. The models can be implemented in a clinical database so that model prognoses for both endpoints are immediately available after patient data have been entered in the database. Currently, we plan to externally validate the model with other University Eye Clinics. Creating an open access form of the model is currently under discussion. 

Of course, we do not screen for all known sight-threatening diseases but for one major cause of visual impairment. Therefore, we also consider personal responsibility and suggest that subjects should be screened anyway due to the risk of contracting other eye-threatening diseases. We suggest that the remaining patients without a prediction should be screened more closely. 

### 4.5. Outlook and Further Developments

We have many ideas for further improving the models in the future: First, we plan to test and analyze more candidate predictor variables based on image data, e.g., GDx^®^, OCT^®^, HRT II^®^, or other structural imaging methods. Modern deep learning methods based on convolutional neural networks (CNNs), such as Inception net, VGGNet, ResNet, R-CNN+LSTM models, or classification maps, have already demonstrated utility for assessing various disease processes, including cataracts, glaucoma, age-related macular degeneration, and diabetic retinopathy [4,18,19,20,21,22]. Of course, these methods should also be applied, especially in combination with clinical data, for predicting the above-addressed endpoints.

Another option is to establish prediction models for subgroups of OAG or related diseases. Moreover, we could ask ourselves if we should increase or decrease the follow-up time (e.g., to 5 or 15 years). Here, we provide models for assessing the development of the most common sub-form of glaucoma (OAG), which is only one of the major reasons for the irreversible impairment of visual function and blindness worldwide. Applying our approach to the other subgroups of glaucoma and other major diseases causing irreversible impairment may reduce the burden of routine screenings tremendously. 

### 4.6. Strengths and Limitations of This Study

#### 4.6.1. Strengths

The most important strength of this study is that it provides methods for safely excluding 43% of all eyes during the initial examination and correctly predicting OAG freeness and freeness from IOP-lowering therapy for 10 years. This significantly decreases the practical screening workload in daily work. Another strength is that the models are not only theoretical concepts/ideas/suggestions, but they can also easily be implemented in any database and then be applied in daily practice. The large sample size of about 600 eyes is also an important strength of this study. A large sample size is important for creating with statistically sound models, as emphasized in mathematical statistics and machine learning theory. It is important to train and independently validate the models with large samples to avoid overlearning and to provide accurate predictions when the models are confronted with new, previously unseen data (generalization). Another strength of this study concerns the sample: right and left eyes were carefully distinguished and were not merged. This would artificially increase the sample size, but in that case, data independence may be undermined. In this case, prediction models may struggle to predict new, previously unseen data. 

#### 4.6.2. Limitations

There might be a possible imbalance between positive and negative groups in terms of predictive performance. This might be due to the fact that optic nerve head measurements were made using TopSS^®^ (Fa. LDT, San Diego, CA, USA), which is outdated. We think that using predictors with higher predictive power (e.g., genetic markers) or using modern topographic measurements of the optic nerve head might improve predictions. Additionally, we recommend conducting follow-up studies with larger samples and evaluating the model with external independent data. However, for this study with such a long FU over 10 years, it was necessary to use this method to achieve a sufficiently large sample size. As described in the Materials and Methods section, visual field examinations were carried out using various different methods. In large-cohort studies such as ours, this is a common drawback over a long study period of more than 20 years, which implies that no uniform data source for the visual field was available. We strongly support using the above procedure for only examining cases where a shallow chamber is observed, as the vast majority of ophthalmologists in practice perform gonioscopy only in cases of a suspected configuration of the anterior segment. When comparing the results of different glaucoma screening programs, it is important to consider to which population the results finally refer. It is important to point out that data for these models were sampled from a European sample. It is well known that ethnicity and race are risk factors for glaucoma, and, therefore, we suggest applying these models in Caucasian patients only.

## 5. Conclusions

Overall, 43% of all eyes can safely be excluded from a glaucoma screening program for up to 10 years while remaining certain that the eyes remain OAG-free and will not need IOP-lowering therapy. We suggest applying this model to inform patients of their future eye health 10 years ahead and to safely exclude the identified eye from further screening within this period. This has a positive impact on reducing the enormous working and screening workloads of ophthalmologists. We suggest applying these neural network models for screening purposes to decide which eyes can be safely excluded and which eyes should be followed-up more closely and sent to a glaucoma expert. This filtering system and identification method will reduce the time and healthcare budgets required for glaucoma screening in the future.

## Figures and Tables

**Table 1:** Univariable odds ratios and descriptive statistics of various predictors for composite endpoint E1: freeness of. OAG and having no IOP-lowering therapy within 10 yrs.

	OAG-Free and no IOP Lowering Therapy 10 yr FU (n = 544)		OAG or IOP-Lowering Therapy at 10 yr FU (n = 41)			
	Mean	Std	Mean	Std	Odds Ratio	*p*-Value
Age (yrs)	58.84	7.96	60.7	7.29	1.02 (0.99–1.08)	0.16 ^1^
IOP (mmHg)	15.1	2.83	18.5	4.27	1.33 (1.21–1.46)	<0.0001 ^1,^*
PEX	12/532	2.2%	3/38	7.3%	3.5 (0.6–13.7)	0.027 ^2,^*
Effective Area (mm^2^)	0.95	0.40	1.08	0.32	2.37 (1.02–5.5)	0.042 ^1,^*
Neuroretinal Rim Area (mm^2^)	1.29	0.32	1.19	0.18	0.38 (0.13–1.09)	0.07 ^1^
Half Depth Area (mm^2^)	0.37	0.21	0.44	0.20	4.0 (1.01–15.8)	0.047 ^1,^*
Half Depth Volume (mm^3^)	−0.06	0.06	−0.07	0.05	0.08 (0.008–7.09)	0.26 ^1^
Volume Below (mm^3^)	−0.25	0.18	−0.31	0.20	0.23 (0.05–0.996)	0.047 ^1,^*
Cup-To-Disc Ratio/0.1 unit change	0.42	0.14	0.48	0.12	1.46 (1.11–1.92)	0.006 ^1,^*

^1^ Independent t-test ^2^ Fisher’s Exact test (one sided), odds ratios are based on univariable logistic regression models, *: indicates statistical significance.

**Table 2:** Overview of baseline demographic data of n = 585 subjects with complete data at baseline and 10 year follow-up ^1^.

	Mean	SD	−95% CI	+95% CI
Age (y)	58.9	8.1	58.8	59.6
Follow-up (years)	11.1	1.1	10.99	11.2
IOP (mmHg)	15.3	3.2	15.1	15.6
Total Contour Area (mm^2^)	2.23	0.44	2.20	2.27
Effective Area (mm^2^)	0.96	0.39	0.93	0.99
Neuroretinal Rim Area (mm^2^)	1.28	0.33	1.25	1.31
Half Depth Area (mm^2^)	0.38	0.21	0.36	0.40
Half Depth Volume (mm^3^)	−0.06	0.057	−0.066	−0.057
Volume Below (mm^3^)	−0.26	0.19	−0.27	−0.24
Cup-To-Disc Ratio	0.42	0.14	0.41	0.43
	**N**	**Percentage**	**−95% CI**	**95% CI**
Endpoint E1: OAG ^2^ at 10-year FU or IOP lowering therapy within 10 years	41/585	7%	5.1%	9.4%
Endpoint E2: OAG ^2^ at 10-year FU	21/585	3.6%	2.23%	5.43%

^1^ All results refer to right eyes. ^2^ OAGs included 6 primary open angle glaucoma (1.03%), 3 pseudoexfoliation glaucoma (0.51%), 0 pigmentary glaucoma (0%), and 12 normal tension glaucoma (2.05%).

**Table 3:** Results of six machine learning algorithms to predict endpoint E1 based on accuracy. Results are given before application of the reject option and are based on the 10-fold cross-validation. Similar results were found for endpoint E2.

Model Performance	Support Vector Machine	Nearest Neighbors	Random Forest	Bayes Classifier	Neural Network
(NPV/PPV)%	93.2/92.3%	93.2/92.3%	93.2/92.4%	90.4/85.5%	93.5/93.2%

After application of the reject option, the best neuronal network model achieved a NPV of 100%, but rejected 314 of 585 (53.6%) patients in the overall sample and similar results were found for endpoint E2 (Table 4).

**Table 4:** Overview performance, advantages and disadvantages of two predictive neural network models with endpoints E1 ^1^ and E2 ^2^.

Endpoint	Percentage of Eyes Filtered Out/Received a Prediction (%)	Performances of the Models ^3^ (n/%)	Advantages	Disadvantages	Remarks
E1 ^2^	57%/43%	253/253 (100%)	In total, 43% of all eyes can safely be excluded from a glaucoma screening program for up to 10 years if one wants to be certain that the eye remains OAG-free and will not require IOP-lowering therapy. This also significantly reduces the screening amount.	57% did not receive a prediction.	Eyes can safely be excluded from the OAG screening program, especially if the patient wants to leave the screening program.
E2 ^1^	53.6%/46.4%	271/271 (100%)	Overall, 46.4% of all eyes can safely be excluded from a glaucoma screening program for up to 10 years if one wants to be certain that the eye remains OAG-free. This significantly reduces the screening workload of ophthalmologists.	Overall, 53.6% did not receive a prediction. Eyes receiving IOP-lowering therapy were not filtered out.	This model is designed for patients in a glaucoma screening setting within a hospital, assuming these patients want to remain within the screening program and continue with IOP-lowering therapy.

^1^ Endpoint 1 (E1): remaining OAG-free within 10 years. ^2^ Endpoint 2 (E2) composite endpoint: remaining OAG-free + no IOP-lowering therapy within 10 years of FU. ^3^ Performances measured in the training and test sample based on neural networks.

**Table 5:** Illustration of prediction model for endpoint E1 based on real data at the initial exam, predicted and observed endpoints at. 10-year follow-up. Identified eyes (green) received a prediction by the model and can safely be excluded from the screening program. Eyes without a prediction (gray) should be send to a glaucoma expert. Patients who suffered of OAG or needed IOP lowering therapy are highlighted in red. No predictions were done in these cases. 332 eyes (57%) were filtered out and did not receive a prediction, 253 (43%) of all eyes received a prediction. 253 out of 253 eyes were correctly predicted (100%). Most importantly, no eye was predicted false-negatively.

Initial Exam									Predictions of Endpoint E1 Made at Initial Exam	Observed Endpoint E1 at the 10 yr FU
Age	IOP ^1^	EA ^2^	NR ^3^	VB ^4^	HDA ^5^	HD ^6^	C/D ^7^	PEX ^8^		
62	12	0.85	1.22	−0.15	0.19	−0.02	0.41	No	No prediction	OAG-free/no IOP low. therapy
63	14	0.66	1.46	−0.15	0.24	−0.03	0.31	No	OAG-free/no IOP low. therapy	OAG-free/no IOP low. therapy
56	15	1.49	1.18	−0.35	0.60	−0.07	0.56	No	OAG-free/no IOP low. therapy	OAG-free/no IOP low. therapy
61	15	1.22	0.76	−0.52	0.48	−0.11	0.62	No	No prediction	OAG
66	14	1.41	1.10	−0.34	0.50	−0.09	0.56	No	OAG-free/no IOP low. therapy	OAG-free/no IOP low. therapy
42	17	0.51	1.73	−0.10	0.19	−0.03	0.23	No	OAG-free/no IOP low. therapy	OAG-free/no IOP low. therapy
54	14	0.85	0.91	−0.13	0.32	−0.02	0.49	No	No prediction	OAG
69	13	0.96	1.14	−0.18	0.39	−0.05	0.46	No	OAG-free/no IOP low. therapy	OAG-free/no IOP low. therapy
66	14	1.13	1.21	−0.31	0.34	−0.06	0.48	No	OAG-free/no IOP low. therapy	OAG-free/no IOP low. therapy
70	14	1.09	1.44	−0.17	0.27	−0.028	0.43	Yes	No prediction	IOP low. therapy
59	18	0.83	1.18	−0.21	0.22	−0.04	0.41	No	OAG-free/no IOP low. therapy	OAG-free/no IOP low. therapy
62	15	0.99	1.21	−0.45	0.39	−0.12	0.45	No	No prediction	OAG-free/no IOP low. therapy
57	12	0.50	1.39	−0.06	0.20	−0.01	0.27	No	OAG-free/no IOP low. therapy	OAG-free/no IOP low. therapy
58	13	1.16	0.97	−0.65	0.66	−0.22	0.54	No	No prediction	OAG-free/no IOP low. therapy
57	16	1.25	0.94	−0.41	0.69	−0.12	0.57	No	No prediction	OAG-free/no IOP low. therapy
62	12	1.21	1.25	−0.64	0.70	−0.19	0.49	No	No prediction	OAG-free/no IOP low. therapy

^1^ Intraocular eye pressure (mmHg), ^2^ effective area (mm^2^), ^3^ neuroretinal rim area (mm^2^), ^4^ volume below (mm^3^), ^5^ half depth area (mm^2^), ^6^ half depth volume (mm^3^), ^7^ cup-to-disc area, ^8^ pseudoexfoliation syndrome.

## Data Availability

Data are available on request due to legal restrictions.

## Supplementary Materials

The following supporting information can be downloaded at: https://www.mdpi.com/article/10.3390/jpm15080361/s1, Figure S1: An illustration of the network architecture with all layers and activation functions; Table S1: An overview about pre-processing, model training, split of data into training, validation and test sample, training stop criteria, selection of thresholds for the reject option and final test of the models based on neural networks; Table S2: Overview of model performances with various combinations of cut-offs for the neural network model with endpoint E1.
