# Supplementary material for: Two Machine Learning Models to Economize Glaucoma Screening Programs: An Approach Based on Neural Nets
Source: J Pers Med. 2025 Aug 7;15(8):361. doi: 10.3390/jpm15080361 (PMC12387565; doi:10.3390/jpm15080361)
Supplement: Supplementary file 1 [file jpm-15-00361-s001.zip › jpm-3603643-supplementary.pdf]

Supplementary Materials

Table S1. An overview about pre-processing, model training, split of data into training, validation and test sample, training stop criteria, selection of thresholds for the reject option and final test of the models based on neural networks.

	Description		***Remarks***
Encoding and input normalization	Input variables of the machine learning models were normalized so that each input has mean 0 and variance 1.	Categorical variables were encoded using one hot encoding	-
Description of training, validation and test samples	Full sample (n=585) was randomly split into a training sample (n=486) and test sample (n=117). 10-fold cross-validation was used for model training. No data of the test sample was used for model training.	Two thresholds for the reject option were selected to maximize negative and positive predictive values and to minimize number of unpredicted cases.	The selected thresholds were evaluated in the training sample and also evaluated in the independent and randomly selected test sample (Tab. 4)
Model training	Adaptive moment estimation were used as stochastic gradient descent method.	-	-
Training stopping criteria	Based on the network loss with a relative change of loss	Maximal training rounds <= 500 rounds	To mitigate overfitting, early stopping approach and 10-fold cross-validation were used. L2-regularization techniques were also used.
Model performances	Overview performance, advantages and disadvantages of two predictive machine learning models with endpoints E1[1] and E2[2] is given in Tab.4.		
Final test	Trained neural network models with two thresholds allowing the reject option were independently tested by evaluating negative, positive predictive power and percentage of unclassified subjects in the training and test sample. Results demonstrate that no overfitting occurred (Tab.4 and Tab. S2)		

Table S2: Overview of model performances with various combinations of cut-offs for the neural network model with endpoint E1.

		**Training and validation sample**			**Test sample**			
**Lower cut-off c1**	**Upper cut-off c2**	**Negative predictive value (NPV) (%)**	**Positive predictive value (PPV) (%)**	**Percentage of unpredicted cases (PUC) (%)**	**Negative predictive value (%)**	**Positive predictive value (%)**	**Percentage of unpredicted cases (PUC) (%)**	**Remarks**
0.5	0.5	432/432 92.9%	3/3 100%	0%	112/117 9 98.7%	n.d.	0%	This corresponds to a model without using a reject option.
0.1	0.9	414/436 95.0%	n.d.	6.8%	108/110 98.1%	n.d.	6.0%	NPV increased in the test sample, PPV was not defined in test sample. PUC increased slightly.
0.01	1.0	260/262 99.1%	n.d.	44%	74/74 100%	n.d.	36.8%	NPV and PUC considerably increased in both training and test sample, PPV was still not defined.
0.0067	1.0	193/193 100%	n.d.	58.7%	60/60 100%	n.d.	48.7%	Final decision about the cut-offs and PUC was done after intensive discussions with the

	medical experts about their suggestions concerning NPV and PPV.

NPV and PUC were 100% and 57% for endpoint E1, respectively after application of the reject option in the total sample (this corresponds to the model with reject option, Tab. 4). The cost for this improvement is that 57% of all patients in the total sample did not receive a prediction for endpoint E1. Both selected cut-offs c1 and c2 were applied in a test sample to check whether results remain stable (Tab. 4).

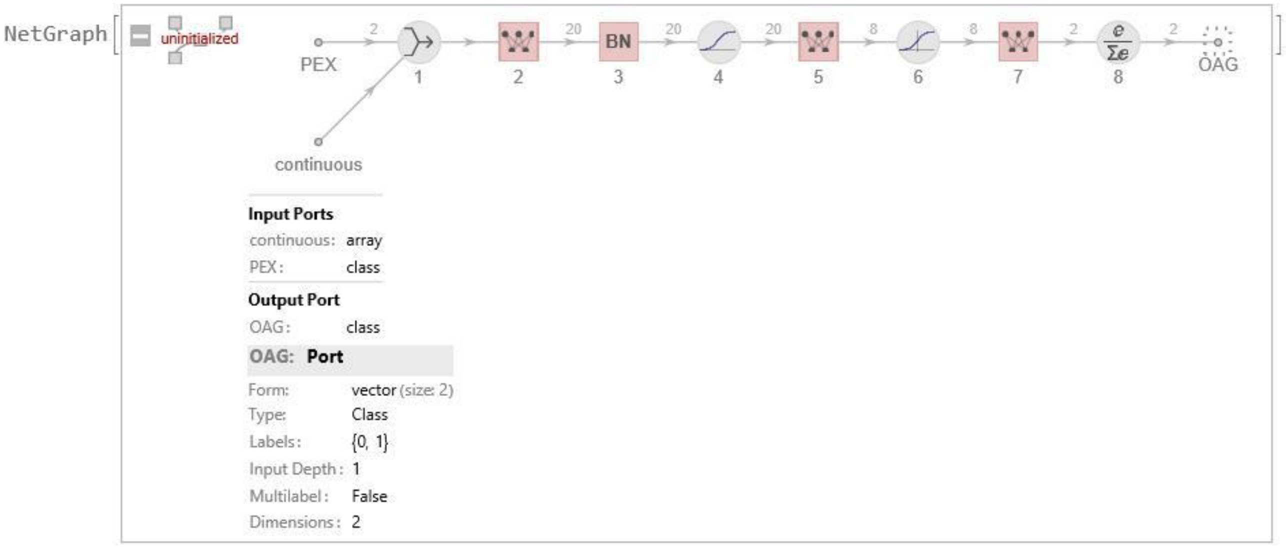

Figure S1: An illustration of the network architecture with all layers and activation functions. Layers and activation functions are enumerated in the figure (1-11): 1) Catenation layer: Input vector consists of 1 discrete (PEX) and 8 continuously distributed variables which were catenated by a catenate layer 2) Linear layer, i.e. a real matrix 3) Batch normalization layer, 4) Logistic sigmoid as used activation function 5) Linear layer 6) Logistic sigmoid as activation function 7) Linear layer 8) Logistic sigmoid as activation function 9) Softmax layer. This network architecture was used for both endpoints.
